# Impact of implementation of polymerase chain reaction on diagnosis, treatment, and clinical course of Acanthamoeba keratitis

**DOI:** 10.1007/s00417-023-05993-7

**Published:** 2023-02-16

**Authors:** Mathias Roth, Adriana Balasiu, Loay Daas, Christoph Holtmann, Anna Servera, Marcus Walckling, Colin R. MacKenzie, Thomas A. Fuchsluger, Gerd Geerling

**Affiliations:** 1 Department of Ophthalmology, Heinrich-Heine University Duesseldorf, Moorenstr 5, 40225 Duesseldorf, Germany; 2 Institute of Medical Microbiology and Hospital Hygiene of the University Hospital Duesseldorf, Duesseldorf, Germany; 3 Department of Ophthalmology, Saarland University Medical Center, Homburg, Germany; 4 Department of Ophthalmology, University Medical Center, Rostock, Germany

**Keywords:** Acanthamoeba, Keratitis, Infectious keratitis, Polymerase chain reaction, PCR, Contact lens

## Abstract

**Purpose:**

Acanthamoeba keratitis (AK) is a painful and possibly sight-threatening ocular infection. While the correct diagnosis and specific treatment in the early stages significantly improve the prognosis, the disease is often misdiagnosed and in clinical examination confused with other forms of keratitis. Polymerase chain reaction (PCR) for the detection of AK was first introduced in our institution in December 2013 to improve the timely diagnosis of AK. The aim of this study was to assess the impact of implementation of Acanthamoeba PCR on the diagnosis and treatment of the disease in a German tertiary referral center.

**Patients and methods:**

Patients treated for Acanthamoeba keratitis between 1st of January 1993 and 31st of December 2021 in the Department of Ophthalmology of the University Hospital Duesseldorf were identified retrospectively via in-house registries. Evaluated parameters include age, sex, initial diagnosis, method of correct diagnosis, duration of symptoms until correct diagnosis, contact lens use, visual acuity, and clinical findings as well as medical and surgical therapy by keratoplasty (pKP). In order to assess the impact of implementation of Acanthamoeba PCR, the cases were divided into two groups (before (pre-PCR group) and after PCR implementation (PCR group).

**Results:**

Seventy-five patients with Acanthamoeba keratitis were included (69.3% female, median age 37 years). Eighty-four percent (63/75) of all patients were contact lens wearers. Until PCR was available, 58 patients with Acanthamoeba keratitis were diagnosed either clinically (*n* = 28), by histology (*n* = 21), culture (*n* = 6), or confocal microscopy (*n* = 2) with a median duration until diagnosis of 68 (18; 109) days. After PCR implementation, in 17 patients, the diagnosis was established with PCR in 94% (*n* = 16) and median duration until diagnosis was significantly shorter with 15 (10; 30.5) days. A longer duration until correct diagnosis correlated with a worse initial visual acuity (*p* = 0.0019, *r* = 0.363). The number of pKP performed was significantly lower in the PCR group (5/17; 29.4%) than in the pre-PCR group (35/58; 60.3%) (*p* = 0.025).

**Conclusions:**

The choice of diagnostic method and especially the application of PCR have a significant impact on the time to diagnosis and on the clinical findings at the time of confirmation of diagnosis and the need for penetrating keratoplasty. In contact lens–associated keratitis, the first crucial step is to take AK into consideration and perform a PCR test as timely confirmation of diagnosis of AK is imperative to prevent long-term ocular morbidity.


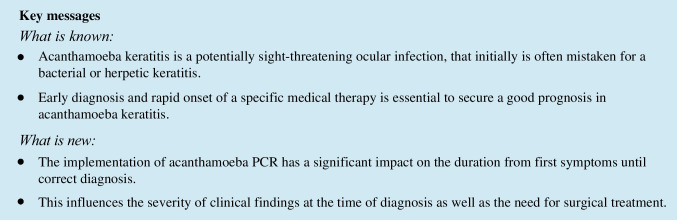


## Introduction

*Acanthamoeba* are opportunistic protozoa, ubiquitously found in the environment. They exist in two forms: under favorable conditions, *Acanthamoeba* remains in the motile trophozoite form, which can transform into a dormant state known as a “cyst” and is highly resistant to adverse conditions, such as extremes in temperature, dryness, and pH, as well as anti-amoebic treatment [1]. Acanthamoeba keratitis (AK) is a painful and potentially sight-threatening ocular infection. With an estimated incidence ranging from 0.33 to 1.49 per 10,000 contact lens wearers or 0.13 to 2.7 cases per million per year in the general population, it is a rare disease [2, 3]. However, an increasing incidence has recently been reported in several countries, probably due to the rising use of soft contact lenses, the predominant risk factor for AK [3–9]. Also, the rising awareness and a shift in diagnostic methods towards PCR and confocal microscopy may have played a role in the rising incidence of AK [3].

While both, diagnosis and specific treatment, in the early stages of disease significantly improve the prognosis of AK, it is often not diagnosed and on clinical examination mistaken for a bacterial or herpetic keratitis. Microbiological detection of *Acanthamoeba* by in vitro cultivation may be difficult, especially as it can be inhibited by prior use of topical drugs with amoebicidal activity and may require prolonged incubation on *Escherichia coli* (*E. coli*). Delayed diagnosis can lead to deeper corneal involvement with severe sequelae requiring prolonged, more intensive, and possibly surgical treatment, including penetrating keratoplasty (pKP) [1, 9, 10]. In prolonged cases with deep infiltrates that are not accessible to corneal scrapings, the clinical diagnosis will sometimes only be confirmed, after a corneal biopsy, e.g., from keratoplasty, is analyzed histologically and/or microbiologically.

The polymerase chain reaction (PCR) is a method of enzymatically amplifying segments of deoxyribonucleic acid (DNA) in vitro. By application of specific primers, mirroring the DNA of, e.g., a microorganism in question, even smallest amounts of this specific DNA can be detected. The use of PCR for the detection of AK was first described in the late 1990s and since then has become more and more important for the diagnosis of AK [11, 12]. In our institution, real-time PCR was first introduced in December 2013 to improve the timely diagnosis of AK. The aim of this study here was to assess the impact of implementation of an Acanthamoeba PCR on the diagnosis and treatment, and clinical course of the disease in a German tertiary referral center.

## Material and methods

Before initiation of the study, approval was obtained from the Ethics Committee of the Medical Faculty of Duesseldorf (file number 4574). The study adhered to the tenets of the declaration of Helsinki. All patients treated for Acanthamoeba keratitis in the Department of Ophthalmology of the University Hospital Duesseldorf between 1st of January 1993 and 31st of December 2021 were identified retrospectively from hospital records. Cases with proof of *Acanthamoeba* (in vitro cultivation, PCR, histology, or in vivo confocal microscopy (IVCM)) and cases without positive microbiological proof but with a clinical diagnosis and positive response to anti-amoebic treatment were included. Patients with clinically suspected Acanthamoeba keratitis but without follow-up, essential data missing, or with clinical improvement without anti-amoebic therapy were excluded.

The following parameters were recorded from paper-based and digital patient records: age, sex, initial diagnosis (defined as diagnosis at the first ophthalmologic presentation regardless of the institution), diagnostic method (defined as method to first establish or confirm the diagnosis of AK), duration of symptoms before correct diagnosis, contact lens use, visual acuity (LogMAR) at the first and last examination, clinical findings (strong pain, epithelial defect, stromal infiltrates, ring infiltrate and hypopyon, perineural infiltrates and pseudodendrites), and medical as well as surgical therapy (pKP). In one patient, both eyes were infected. In this case, the average of the visual acuity was used. In order to assess the impact of implementation of Acanthamoeba PCR, the cases were divided into 2 groups. All cases before PCR were available at our tertiary center (until 16 December 2013) and were evaluated together as pre-PCR group and cases thereafter evaluated together as PCR group. Furthermore, the time from first visit until the PCR result was available and the number of individual patients per year for whom Acanthamoeba PCR was requested was evaluated.

### Statistical evaluation

Statistical analysis was performed using Prism 9.0.0 (GraphPad, La Jolla, CA, USA). The normality of distribution of the data was analyzed with the Shapiro–Wilk test. Data are presented descriptively with the median and interquartile range (IQR) (presented as *median* (*25th percentile*; *75th percentile*). For group comparisons, the Mann–Whitney *U* and Kruskal–Wallis test were performed. Fisher’s exact test, the chi-squared test, and Spearman’s *R* were used to investigate correlations. *p* values ≤ 0.05 were considered statistically significant.

## Results

Between January 1993 and December 2021, 75 patients with Acanthamoeba keratitis that met the inclusion criteria were identified (69.3% female, median age 37 (22; 48) (range 15–73) years). In total, 29 cases were excluded (loss of follow-up: 1; clinical improvement without anti-amoebic therapy: 4; essential data missing/missing records: 24). The median visual acuity at first examination was 0.70 (0.30; 1.70) logMAR, and at last follow-up 0.30 (0.10; 0.70) logMAR. Eighty-four percent (63/75) of all patients used contact lenses, 43 of which were soft and 13 rigid; in 7 cases, no data regarding the type of contact lens was available. Overall, the diagnosis of Acanthamoeba keratitis was established clinically in 28 (37.3%), histologically in 21 (28%), by PCR in 17 (22.6%), by culture in 6 (8%), and by IVCM in 3 cases (4%). Before PCR became available in our department, 58 patients were diagnosed with Acanthamoeba keratitis, of whom a single patient (1.7%) presented with a positive PCR result from an external source. Furthermore, of those 58 patients, the diagnosis was first established clinically in 28 cases (48.2%), established or confirmed by histology in 21 cases (36.2%), by culture in 6 cases (10.3%), and by IVCM in 2 cases (3.4%). After PCR was implemented, the diagnosis was established with PCR in 94% (16/17) of the cases (PCR group: *n* = 17; methods of diagnosis: PCR: *n* = 16/17, IVCM: *n* = 1/17). There was no statistically significant difference regarding age, gender, visual acuity, and contact lens use between the various diagnostic methods as well as the pre-PCR vs. PCR groups (Fig. 2).

Between December 2013 and December 2021, Acanthamoeba PCR was requested in 890 individual patients. The yearly median of requests was 110.5 (72; 150) in the full years 2014–2021, the median of positive PCR results per year was 2 (1; 3). Four cases with positive PCR results in contact lens material were excluded, as they showed rapid clinical improvement without anti-amoebic therapy. However, two cases that also showed positive PCR results only in contact lens material were included based on typical clinical findings, including a ring infiltrate in both cases. In all other cases in the PCR group, *Acanthamoeba* could be detected in corneal samples.

**Fig. 1:**
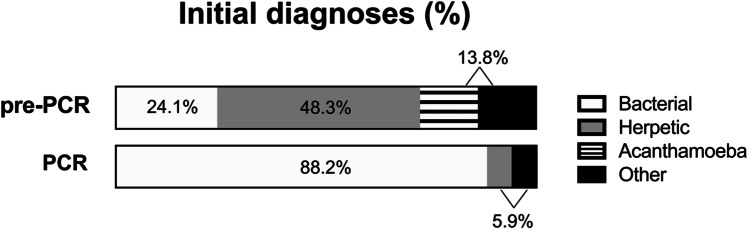
Differences in the initial diagnosis between pre-PCR vs. PCR group. In the pre-PCR group, the correct diagnosis was already established initially in 13.8% vs. 0% in the PCR group. In the PCR group, all cases were initially misdiagnosed, mostly as bacterial keratitis (88.2%)

The initial diagnosis differed significantly between pre-PCR vs. PCR groups. While in the pre-PCR group the correct diagnosis was already established initially in 13.8% (8/58) of the cases, in the PCR group, all initial diagnoses were incorrect with bacterial keratitis dominating with 88.2% (15/17) (vs. pre-PCR 24.1% (14/58); *p* < 0.001). In the pre-PCR group, herpetic keratitis (48.3% (28/58)) was significantly more common as an initial diagnosis than in the PCR group (5.9% (1/17); *p* = 0.002). Figure 1 shows the percentage of the different initial diagnoses in both groups.

**Fig. 2:**
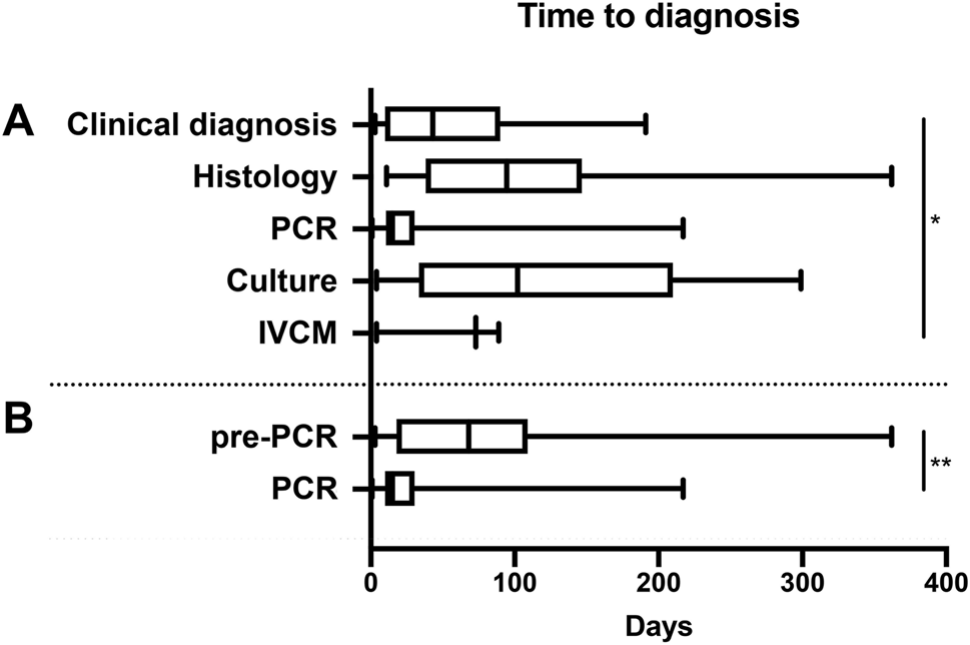
Duration from first symptoms to correct diagnosis. The duration in days between first symptoms until correct diagnosis differs significantly between the diagnostic methods ((**A**) median (IQR) in days: clinical diagnosis: 43 (10; 90); histology: 94.50 (38; 147); PCR: 15 (10.5; 30.5); culture: 102 (33.5; 210); IVCM: 73 (4;89); *p* = 0.0118) as well as between the pre-PCR vs. PCR group ((**B**) median (IQR) in days: pre-PCR: 68 (18; 109); PCR: 15 (10; 30.5); *p* = 0.0061)

The duration from beginning of symptoms until correct diagnosis could not be evaluated in three cases due to missing data. Overall, the duration from beginning of the symptoms until correct diagnosis was 47 (14; 100) days. The time from first visit in our clinic until a positive PCR result was available was 3.5 (2; 7) days. The diagnostic methods as well as the pre-PCR vs. PCR groups differed significantly in the time to establish the correct diagnosis (Fig. 2). While a longer duration until correct diagnosis correlated with a worse initial visual acuity (*p* = 0.0019, *r* = 0.363), the visual acuity between the two groups at last follow-up was not significantly different.

**Fig. 3:**
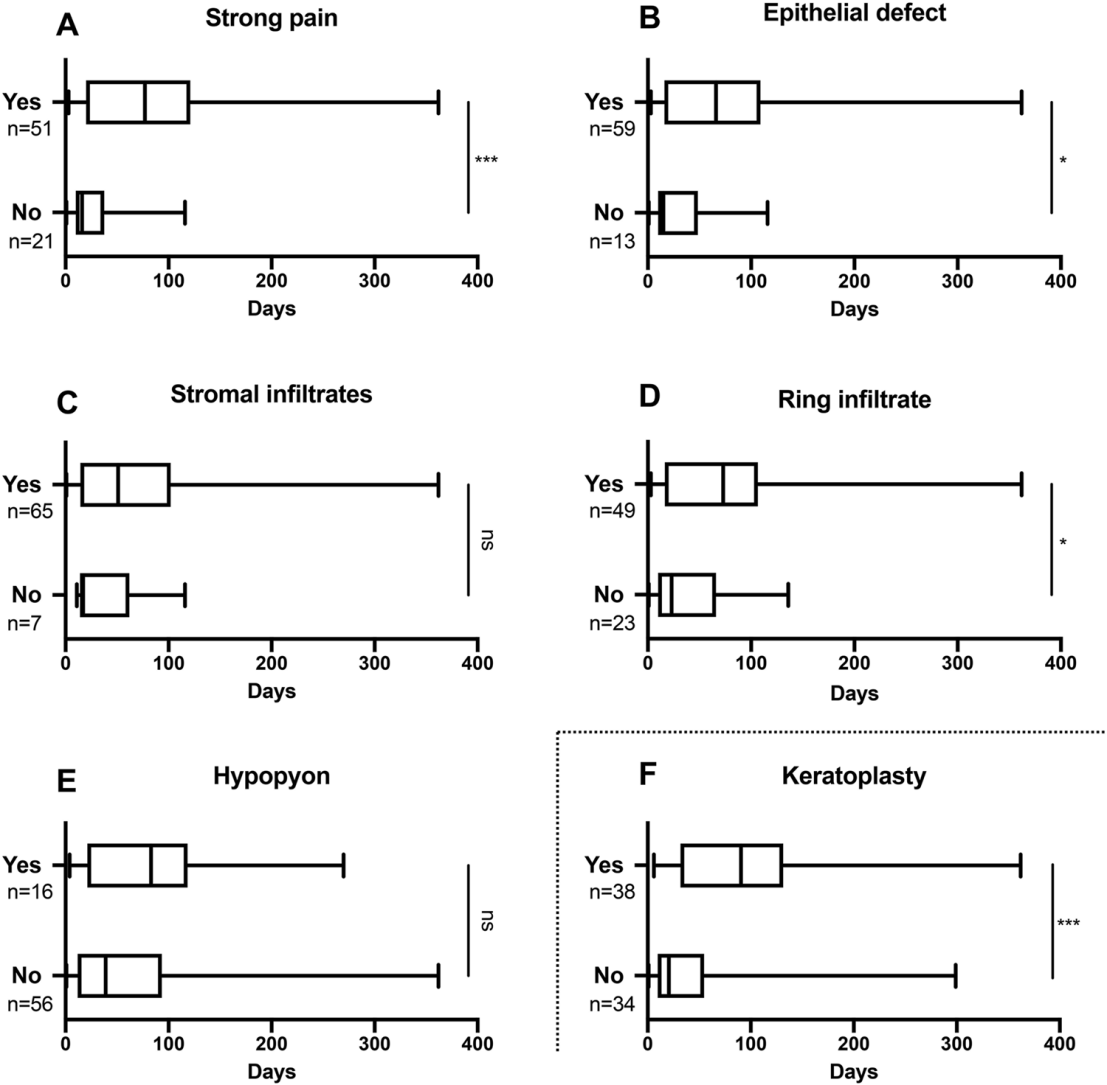
Influence of duration from first symptoms to correct diagnosis on clinical findings and need for pKP. A significant correlation of the appearance of strong pain ((**A**) *p* < 0.001), epithelial defect ((**B**) *p* = 0.017), and ring infiltrate ((**D**) *p* = 0.038) with a longer duration from first symptoms to correct diagnosis was shown. There was no significant correlation between duration to diagnosis and perineural infiltrates as well as pseudodendrites (not shown). (**F**) Furthermore, the number of pKPs performed significantly correlated with a longer duration from first symptoms to correct diagnosis (*p* = 0.0001)

**Fig. 4:**
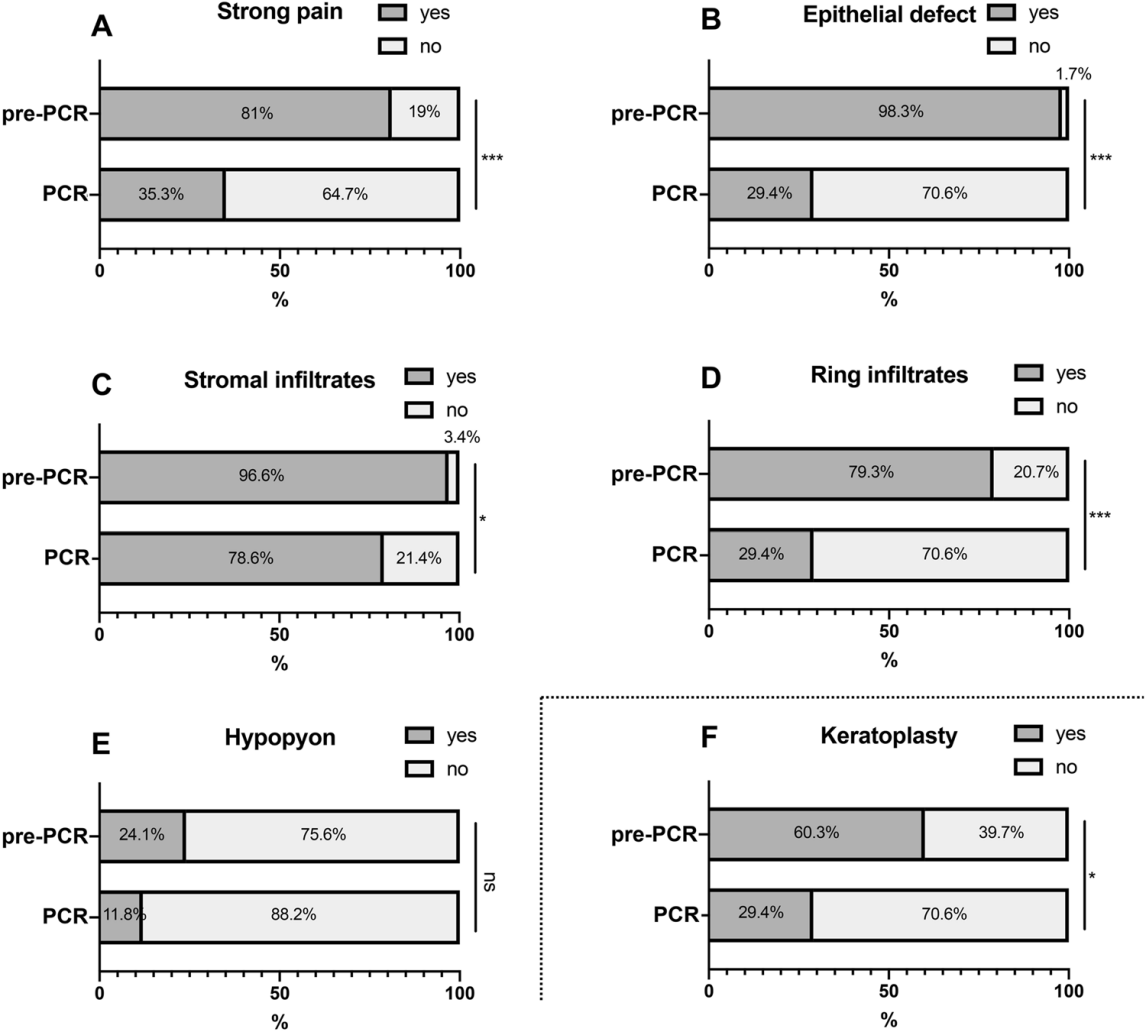
Impact of the use of PCR on clinical findings and need for pKP. Due to the significantly shorter duration from first symptoms to correct diagnosis in the PCR group, also in the group-comparison pre-PCR vs. PCR, the ratio of appearance of strong pain ((**A**) *p* < 0.001), epithelial defect ((**B**) *p* < 0.001), and ring infiltrate ((**D**) *p* < 0.001) is significantly higher in the PCR group. Furthermore, this analysis shows a significant higher ratio of stromal infiltrates in the pre-PCR group ((**C**) *p* = 0.018). Also, in the pre-PCR group, a significantly higher number of patients needed a pKPs ((**F**) *p* = 0.025). There was no significant correlation between duration to diagnosis and perineural infiltrates as well as pseudodendrites (not shown)

The appearance of strong pain, epithelial defect, and ring infiltrate shows a correlation with a longer duration from onset of symptoms until correct diagnosis and thus also with the different diagnostic methods as well as the pre-PCR vs. PCR groups (Figs. 3 and 4).

Overall, the medical therapy in all cases was comparable, consisting of a high frequency application of polyhexamethylene biguanide (PHMB) 0.02% and propamidine isethionate (Brolene ®) 0.1% together with topical antibiotics. In total, a pKP was performed in 53.3% (40/75) of all cases. Overall, 40 pKPs were performed in median 111 (91; 159) days after onset of symptoms. There was no significant difference in the duration between onset of symptoms until surgery between the pre-PCR and PCR group. However, the number of pKPs performed was significantly lower in the cases with a shorter duration from onset of symptoms until correct diagnosis and thus also in the PCR group (5/17; 29.4%) than in the pre-PCR group (35/58; 60.3%) (*p* = 0.025) (Figs. 3 and 4).

## Discussion

Our study shows that the implementation of Acanthamoeba PCR had a significant impact on the time until correct diagnosis and on the severity of clinical findings at the time of diagnosis as well as the need for surgical treatment.

### Duration until correct diagnosis

Early diagnosis and rapid onset of appropriate medical therapy are essential to secure a good prognosis. Delayed therapy worsens the prognosis and increases the likelihood for keratoplasty [13–17]. In our PCR group, the duration from symptoms until initial diagnosis of AK of 15 days is significantly shorter comparable to the literature, as reported, e.g., by Ross et al. to be 27 days [13]. In our department, microbiological test routine includes corneal scraping for smears, culture, and PCR. As PCR results were available at a median of 3.5 days after presentation, this documents the high speed at which this method enables the clinician to establish the diagnosis of AK.

In the study by Ross et al., most diagnoses are confirmed by culture (88%) or IVCM (72%), while PCR was not used [13]. Maybe it was not regularly available yet, as the included cases were from 2008 to 2011. Shah et al. only included culture-proven AK cases and reported a mean time of 53 days from onset of symptoms to culture collection [18]. Data from the German Acanthamoeba Keratitis Registry indicate an exceptionally long delay until correct diagnosis of 2.8 months by a selection bias towards more difficult cases by tertiary referral centers which may also explain the high rate of corneal transplantations and thus histological confirmation (55%) in the registry [1].

### Clinical findings and initial diagnosis

The literature describes several clinical signs as highly indicative of AK, which may however vary as disease progresses [13, 19, 20]. Strong pain, often described as disproportionate to the clinical findings, has long been recognized as a hallmark of AK and is sometimes especially referred to as an early sign of the disease [20]. However, ocular pain presented variably in different studies and absence of pain does not exclude the diagnosis [1, 20, 21]. As our results confirm, it is more often absent in early diagnosed cases and increasingly found with longer duration.

**Fig. 5:**
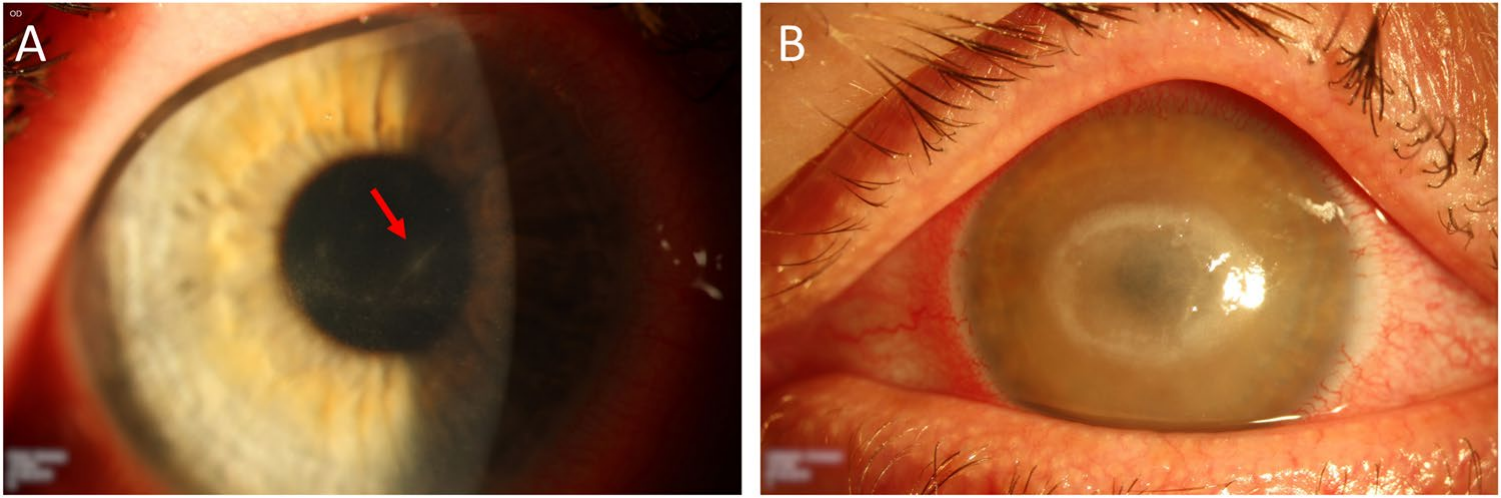
Slit lamp images of clinical findings (**A**) The patient presented at our clinic 3 weeks after onset of symptoms because of increasing pain and a slowly decreasing visual acuity (0,2 LogMAR). Slit lamp examination revealed only slight epithelial changes (gray epithelial opacities, epithelial microerosions and microcysts), known as “dirty epithelium” [19] and a perineuritis (red arrow). (**B**) This patient presented 5 weeks after first symptoms. Visual acuity was 2,0 LogMAR. In the slit lamp examination, a ring infiltrate, a central corneal erosion, and a rough corneal surface were found. (**A**, **B**) Both patients were using soft contact lenses and before presentation at our clinic, they were treated with antibiotics because of suspected bacterial infection. Shortly after presentation at our clinic, Acanthamoeba keratitis was confirmed by PCR in both cases

While the ring infiltrate or Wessely’s immune ring (Fig. 5) sometimes is regarded as almost pathognomonic [22], it is important to note that it is a nonspecific finding that can occur in infectious keratitis of various origin as well as non-infectious etiology and thus can be also misleading [23]. Our data show that a ring infiltrate is rather seen in advanced stages [20, 24, 25]. Also, in keeping with our results, an epithelial defect is rather found late in the disease and was present more often in the pre-PCR group in our study in which diagnosis was further delayed [25]. Probably because of these time-dependent differences in the clinical signs and symptoms, the distribution of initial clinical diagnoses changes, depending on whether the correct diagnosis is established early (PCR group) or late (pre-PCR group) (Fig. 5). In the PCR group, we found a significantly higher rate of suspected “bacterial infection.” In the pre-PCR group, “herpetic keratitis” was the predominant initial diagnosis (48.3%), which is in line with the results of the literature [1, 3, 18, 26, 27].

### Need for keratoplasty

As mentioned earlier, the need for keratoplasty is significantly higher in more advanced cases, i.e., if diagnosis is delayed, e.g., due to an initial misdiagnosis [13–17, 26]. While overall the rate of cases requiring a pKP in our study (40/75; 53.3%) is higher than in the German Acanthamoeba registry (37%), the number of corneal grafts performed was significantly lower in the PCR group (5/17; 29.4%) than in the pre-PCR group (35/58; 60.3%). Bacon et al. also found a positive effect of early diagnosis on the need for corneal transplantation. Out of 15 patients who were treated within 1 month of initial symptoms, only one patient (6.7%) required penetrating keratoplasty [15]. While in advanced stages of the disease, a penetrating keratoplasty is the preferrable treatment option, in early cases unresponsive to medical treatment and depending on the extent of the infection a lamellar keratoplasty might be preferred to avoid the risk of spread inside the eye and a possible endothelial immune rejection [28–31]. If available, anterior segment optical coherence tomography and IVCM should be used to evaluate the depth and extent of the infection [31].

### Comparison of methods

In vitro culture on *E. coli* plates for AK confirmation can take up to several weeks and the sensitivity ranges from 0 to 70% [19]. Due to the crucial importance of an early treatment, this technique is becoming less relevant and has been replaced by PCR in our microbiological department.

Histopathological analysis of corneal material has a sensitivity of 31–65% [19, 25, 32]. Because of the limited amount of specimen available for examination in, e.g., corneal scrapings, this specimen will most probably be used for microbiological rather than histological analysis. Nevertheless, in intermediate and late cases, repeated scrapings or even a trephine biopsy could be justified for histological evaluation [25].

IVCM is a non-invasive technique that allows real-time visualization of Acanthamoeba cysts in the cornea and can be especially valuable in cases of deep infiltrates not accessible to corneal scrapings and as a control measure during an ongoing treatment [9, 19]. In addition, it can be used for early detection of recurrence [33]. In a recent comparison of diagnostic methods for AK confirmation, the sensitivity of IVCM was higher than PCR (IVCM 77%; PCR 63%) [34]. IVCM is reported to reach a sensitivity of up to 100% [35, 36]. The sensitivity though is very much depending on the experience of the examiner, as cysts can be easily confused for immune cells and as the sensitivity of single IVCM features of AK is low [37–39]. Nevertheless, the longer the duration of AK, the higher the likelihood that a correct diagnosis is made by the observers in grading the IVCM images, probably due to the greater number of Acanthamoeba cysts and a reduction in the type and number of host immune cells [37]. Due to the small field of view (0.2 × 0.2 mm), the application can be time-consuming [40]. But, in near future, automated wide-field montages might facilitate the process of IVCM image acquisition of larger areas of the cornea [41]. In our study, only 4% of the cases were diagnosed with IVCM, in contrast to more than 20% in the German Acanthamoeba registry [1]. Furthermore, the duration to correct diagnosis by IVCM was relatively long in our analysis. Those two results possibly highlight the limitations of IVCM. Dart et al. suggest using the technique in the initial evaluation of cases, but not to make definitive diagnosis if response to amoeba-specific therapy is poor and tissue diagnosis (e.g., PCR) remained negative [20].

In the literature, the sensitivity of Acanthamoeba PCR ranges between 65 and 100% and specificity between 99.3 and 100% [20, 35, 42–45]. In the pre-PCR group in 27 cases, the diagnosis could not be confirmed by any of the described methods but was established clinically by a positive response to anti-amoebic treatment. Since introduction of diagnostic Acanthamoeba PCR in our institution, no case was simply diagnosed based on the clinical picture on presentation or treatment response. PCR is known to significantly increase the confirmation rate of AK in comparison to culture [46], but it may also give a positive result, in the absence of any viable pathogen, simply due to the presence DNA/RNA remnants. It thus cannot be used to assess the activity of an infection [20]. After PCR implementation in our diagnostic workup, the diagnosis of AK was established by PCR in 94% (16/17) and in only 1 additional case by another modality, i.e., IVCM. Six months after PCR as a rapid and sensitive method for the diagnosis of Acanthamoeba keratitis became available at out institution, with lower turnaround time costs than traditional techniques, routine use of culture for Acanthamoeba diagnostics was ceased.

The number of PCR analyses conducted in 8 years (890) may seem very high in relation to a total of 17 *Acanthamoeba* cases diagnosed by this method (positivity rate: 1.9%). PCR is mostly ordered in infectious keratitis not responding to the antibiotic therapy already initiated, and/or presence of clinical findings and/or risk factors (mostly contact lens use) that could be indicative of *Acanthamoeba* or fungi. For this reason, and additionally to bacterial culture, PCR for *Acanthamoeba* is often performed together with PCR for fungi and vice versa. Thus, the high number of conducted PCR analyses reflects all cases of infectious keratitis with unknown etiology, rather than just suspected Acanthamoeba keratitis.

The different diagnostic methods for AK differ not only in the prerequisite of every center, as well as the time until the results are available, but also strongly in the cost per examination. A cost analysis of the different methods might be the focus of future research.

### Other free-living amoebae as pathogen

Currently, almost exclusively *Acanthamoeba* are in the focus in suspected protozoan keratitis. But—although discussed controversially [47–50]—also other free-living amoebae (FLA) as *Hartmannella* or *Vahlkampfia* seem to have the ability to lead to an opportunistic corneal infection that can be clinically indistinguishable from an Acanthamoeba infection [51–56]. Fortunately, these infections seem to respond well to standard anti-amoebic treatment [51–54]. Thus, in cases of presumed amoebic keratitis and when *Acanthamoeba* cannot readily be cultured or identified, as in 28 patients of our analysis, those other FLA should be considered and microbiologically tested for. Irrespective of the amoebic species causing keratitis, early diagnosis and proper anti-amoebic treatment are crucial to yielding a cure.

### Limitations

Our study has some limitations. Being a retrospective case series, with necessary reliance on accuracy and completeness of the clinical records, our findings are limited by potential documentation bias. Furthermore, we want to point out that the data of the study are strictly related to the routine of our center and thus cannot be universally valid.

## Conclusions

Our results show that the choice of diagnostic method and especially the application of PCR have a significant impact on the time to diagnosis and on the clinical findings at the time of confirmation of diagnosis and the need for penetrating keratoplasty. Nevertheless, PCR and/or confocal microscopy are typically not available in primary eye care settings [57]. Thus, especially in every contact lens–associated keratitis, patients should be referred to a tertiary center, to timely confirm or exclude the diagnosis of AK. ﻿
